# Nomogram model for predicting cause-specific mortality in patients with stage I small-cell lung cancer: a competing risk analysis

**DOI:** 10.1186/s12885-020-07271-9

**Published:** 2020-08-24

**Authors:** Jianjie Li, Qiwen Zheng, Xinghui Zhao, Jun Zhao, Tongtong An, Meina Wu, Yuyan Wang, Minglei Zhuo, Jia Zhong, Xue Yang, Bo Jia, Hanxiao Chen, Zhi Dong, Jingjing Wang, Yujia Chi, Xiaoyu Zhai, Ziping Wang

**Affiliations:** 1 Key Laboratory of Carcinogenesis and Translational Research (Ministry of Education/Beijing), Department of Thoracic Medical Oncology, Peking University Cancer Hospital & Institute, 52 Fucheng Road, Haidian District, Beijing, 100142 China; 2 Department of Epidemiology and Biostatistics, School of Public Health, Peking University, Beijing, China

**Keywords:** SCLC, Competing risks, Cumulative incidence, Nomogram

## Abstract

**Background:**

The five-year cumulative incidence rate in patients diagnosed with stage I small-cell lung cancer (SCLC) who were instructed to undergo surgery was from 40 to 60%.The death competition influence the accuracy of the classical survival analyses. The aim of the study is to investigate the mortality of stage I small-cell lung cancer (SCLC) patients in the presence of competing risks according to a proportional hazards model, and to establish a competing risk nomogram to predict probabilities of both cause-specific death and death resulting from other causes.

**Methods:**

The study subjects were patients diagnosed with stage I SCLC according to ICD-O-3. First, the cumulative incidence functions (CIFs) of cause-specific death, as well as of death resulting from other causes, were calculated. Then, a proportional hazards model for the sub-distribution of competing risks and a monogram were constructed to evaluate the probability of mortality in stage I SCLC patients.

**Results:**

1811 patients were included in this study. The five-year probabilities of death due to specific causes and other causes were 61.5 and 13.6%, respectively. Tumor size, extent of tumor, surgery, and radiotherapy were identified as the predictors of death resulting from specific causes in stage I SCLC. The results showed that surgery could effectively reduce the cancer-specific death, and the one-year cumulative incidence dropped from 34.5 to 11.2%. Like surgery, chemotherapy and radiotherapy improved the one-year survival rate.

**Conclusions:**

We constructed a predictive model for stage I SCLC using the data from the SEER database. The proportional sub-distribution models of competing risks revealed the predictors of death resulting from both specific causes and other causes. The competing risk nomogram that we built to predict the prognosis showed good reliability and could provide beneficial and individualized predictive information for stage I SCLC patients.

## Background

Small-cell lung cancer (SCLC) is one of the two main types of lung cancer with short doubling time, high malignancy, and early and extensive metastasis, accounting for approximately 15% of the lung malignancies. SCLC is sensitive to radiotherapy and chemotherapy but highly prone to drug resistance and relapse. The incidence of SCLC is 6.0 per1000,000 persons [1], and the five-year survival rate is 7%. Because of the pathophysiological characteristics of SCLC, a vast majority of patients have been diagnosed with lymph nodes or distant metastases and lost indications for surgical treatment. Patients with stage I SCLC were recommended to take surgery and postoperative chemotherapy according to National Comprehensive Cancer Network (NCCN) Clinical Practice Guidelines in Oncology (version 2.2018) [2].

Survival analyses are common statistical analysis methods in prognosis research; however, classical survival analyses generally deal with only one type of event, which the researchers are interested in, for example, relapse. Many SCLC patients ultimately die from other diseases instead of lung cancer, indicating that there are death competition causes in SCLC; therefore, it is necessary to use a competing risk regression model when evaluating the prognosis of SCLC. In the presence of competing risks, the classical survival analyses are inaccurate because we cannot assume that the follow-up period is sufficiently long for the event we care about to occur. Nomograms are statistical models, and the basic principle of nomograms is to provide the score of each influencing factor according to the contribution degree of each influencing factor in the regression model, and then, calculate the total score of an individual, so as to obtain the predicted value of the individual.

In this study, we aimed to evaluate the effects of the competing causes for the SCLC survival rate and to establish a competing risk nomogram to quantitatively analyze the survival differences in SCLC patients.

## Methods

### Study population

The data on patients with stage IA and IB small cell lung cancer (SCLC) were obtained from the SEER database (2004–2014) using SEER*Stat (v8.3.2). The study cohort consisted of the patients with the following International Classification of Diseases for Oncology Third Edition (ICD-O-3), morphology codes: 8002/3; 8041/3, 8042/3, 8043/3, 8044/3, and 8045/3; and the site codes: C34.0, C34.1, C34.2, C34.3, C34.8, and C34.9. The exclusion criteria were as follows: (1) age at diagnosis less than 18 years, (2) dead or without pathological information, and (3) lack of complete epidemiology and clinical information.

The demographic and clinical pathological data included age, gender, race, anatomical site, laterality, tumor size, tumor degree, grade, and treatment forms. Race was divided into black, white, and others. Three groups were formed according to age (less than 60 years, 60–75 years, and more than 75 years). The anatomic sites were divided into upper, middle, lower, bronchus, and others. Laterality included left and right. The extent of tumor was divided into local and regional, and the grading was classified as good, moderate, poor, undifferentiated, and NOS. The forms of treatment were surgery, chemotherapy, and radiotherapy. The complete SEER session information was added to a supplemental document.

### Statistical analysis

The primary end-point of the study was cause-specific mortality. According to the cause of death (COD) code, we classified the cause of death as cancer-specific death and death resulting from other causes. The covariates added to the model were mainly selected from the available clinically prognostic factors recorded in the SEER database. The covariates included were gender, age, race (black, white, or others/unknown), anatomic sites (upper, middle, lower, bronchus, or others), laterality (left or right), tumor size, extent of tumor (local or regional), grading (good, moderate, poor, undifferentiated, or NOS), chemotherapy (yes or no), radiotherapy (yes or no), and surgery (yes or no). For describing the probability of death, we chose the cumulative incidence function (CIF) and Gray’s test [3]. Ages at diagnosis were regrouped as follows: less than 60 years, 60–75 years, and more than 75. Tumor sizes were grouped into three categories: ≤3 cm, 3–5 cm, and > 5 cm.

We adopted the Fine and Gray proportional hazards model to assess the three- and five-year probabilities of the two competing mortality events [4]. The restricted cubic splines with three empirical knots (10, 50, and 90%) were fitted to the model [5]. Gray’s test was used to compare the difference in the CIF between the two different outcomes. Backward stepwise selection based on Bayesian Information Criterion was used to further eliminate redundant variables. The resulting multivariate Cox regression model was used to calculate risk score and build the final nomogram prognostic model. The Harrell C index5 was applied to indicate the discrimination, and the calibration plot obtained using the method provided by Gray [3] was adopted to evaluate the calibration [6, 7]. Both discrimination and calibration were assessed by bootstrapping with 1000 resamples.

All the statistical analyses were carried out with the R software (v3.3.3). The R packages cmprsk [8], mstate [9] and rms [10] were used for modeling and developing the nomogram. All the reported significance levels were two-sided, and the *P* value for statistical significance was defined as *P* < 0.05.

## Results

### Patient characteristics

**Fig. 1:**
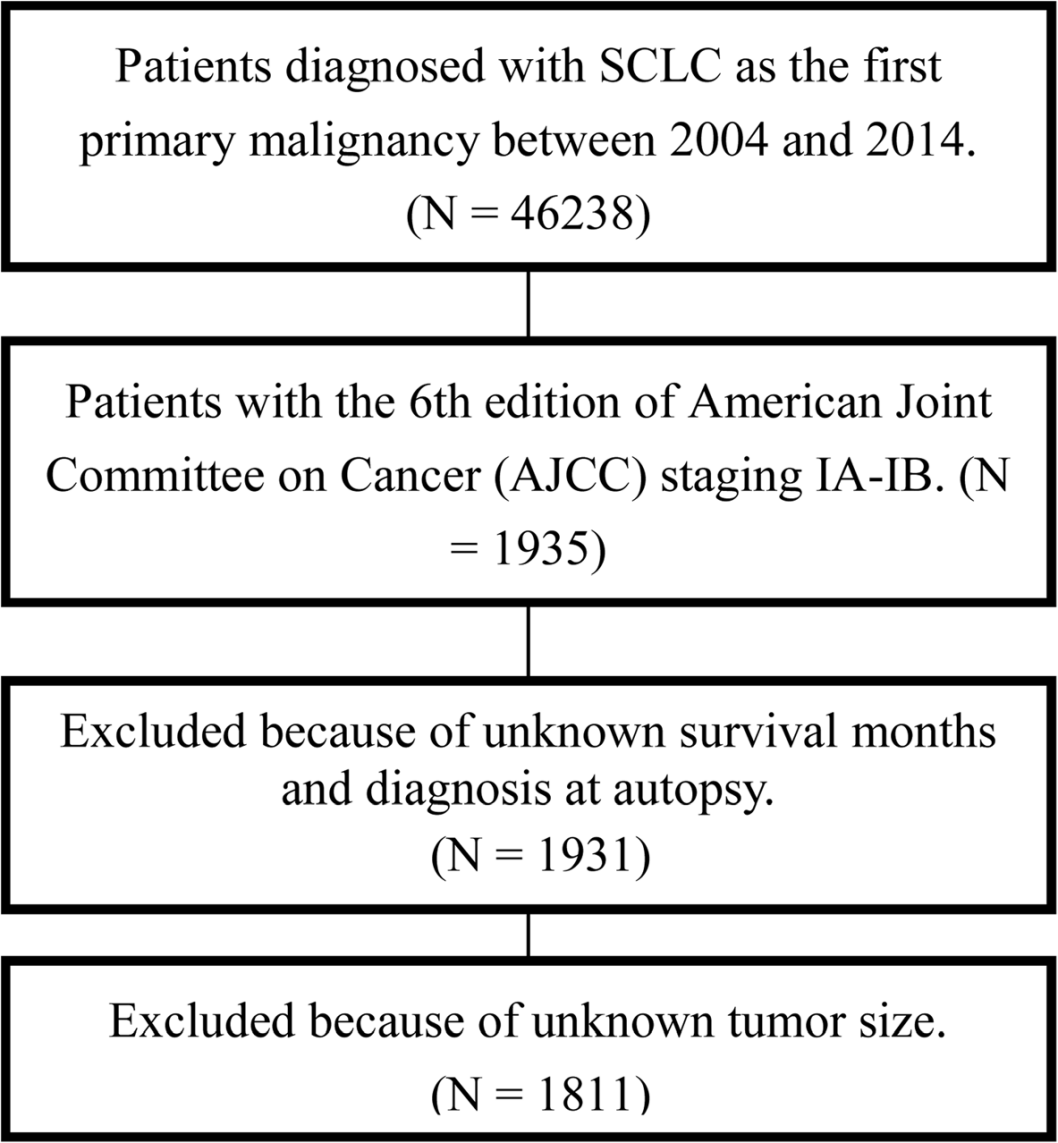
Flow chart showing the process of patient selection. Patients were selected according to several criteria: (1) stage IA-IB, (2) cases with complete information about survival, follow-up months, and cause of death, (3) cases with known tumor size

We selected 1811 eligible stage I SCLC patients (Fig. 1). The distribution of the patients’ demographics and clinical characteristics is presented in Table 1. Of these, 342 (18.9%) patients were aged < 60 years, 981 (54.2%) were aged 60–74 years, and 488 (26.9%) were aged more than 75 years. The number of female patients was 949 (52.4%) and that of the Caucasians was 1578 (87.1%). The most common site was the upper lobe (56.6%), followed by the lower lobe (27.9%) and the other areas (15.56%). The number of patients with a right-sided primary tumor was 1018 (56.2%). The distribution of the tumor size was 53.4, 28.9, and 17.7% for < 3 cm, 3–5 cm, and > 5 cm. As for the tumor extension, the local and the regional ones accounted for 84.3 and 15.7%, respectively. In all, 457 (25.2%) patients were treated with surgery, 929 (51.3%) patients were treated with radiotherapy, and 1217 (67.2%) patients were treated with chemotherapy.

**Table 1:** One-, three-, and five-year cumulative incidence of mortality in stage I SCLC patients

Characteristics	N	%	Event	%	Cancer-specific death				Death from other causes			
					1-year (%)	3-year (%)	5-year (%)	*P*	1-year (%)	3-year (%)	5-year (%)	*P*
Total	1811		1221		28.7	56.5	61.5		5.8	11.1	13.6	
Age at diagnosis								< 0.001				0.001
< 60 years	342	18.9	192	15.7	18.6	49.8	53.1		5.2	7.7	8.2	
60–75 years	981	54.2	640	52.4	26.1	53.5	59.5		5.5	10.7	14.3	
> 75 years	488	26.9	389	31.9	40.9	67.1	71.4		6.9	14.2	15.9	
Gender								0.574				0.016
Female	949	52.4	619	50.7	27.3	56.3	60.5		5.1	9.1	11.3	
Male	862	47.6	602	49.3	30.1	56.7	62.6		6.5	13.3	16.0	
Race								0.690				0.183
White	1578	87.1	1064	87.1	28.9	56.1	60.8		5.9	11.3	13.8	
Black	167	9.2	111	9.1	26.4	57.9	66.0		5.7	7.8	9.9	
Others	66	3.7	46	3.8	27.7	64.3	66.6		3.3	15.1	17.4	
Anatomic sites								< 0.001				0.048
Upper	1025	56.6	685	56.1	26.5	55.9	60.6		5.6	11.8	14.5	
Middle	115	6.4	76	6.2	22.7	51.6	55.1		12.5	19.8	19.8	
Lower	505	27.9	336	27.5	29.4	55.7	61.8		5.1	8.0	11.1	
Bronchus/others	166	9.2	124	10.2	43.6	66.0	70.5		4.5	9.9	10.8	
Primary tumor location								0.221				0.682
Left-sided	793	43.8	522	42.8	28.1	55.3	59.8		6.2	12.0	14.1	
Right-sided	1018	56.2	699	57.2	29.1	57.4	62.8		5.5	10.4	13.1	
Tumor size								< 0.001				0.030
≤ 3 cm	967	53.4	602	49.3	23.7	50.6	55.0		5.7	12.1	15.7	
3–5 cm	523	28.9	379	31.0	30.5	59.6	66.1		7.1	10.9	12.1	
> 5 cm	321	17.7	240	19.7	40.5	68.8	73.3		3.9	8.5	9.5	
Tumor extension								< 0.001				0.005
Local	1526	84.3	1020	83.5	27.9	55.4	59.7		6.3	11.8	14.6	
Regional	285	15.7	201	16.5	32.5	62.2	71.3		3.3	7.3	8.0	
Grading								0.038				0.375
Good or moderate	34	1.9	18	1.5	16.3	36.6	41.3		3.1	6.8	16.7	
Poor	343	18.9	207	17.0	26.3	51.3	54.1		4.9	11.8	13.3	
Undifferentiated	439	24.2	318	26.0	26.5	58.0	65.0		5.6	8.4	11.6	
NOS	995	54.9	678	55.5	30.8	58.1	62.9		6.3	12.3	14.5	
Surgery								< 0.001				0.593
Yes	457	25.2	214	17.5	11.2	35.6	40.4		4.6	9.5	12.0	
No	1354	74.8	1007	82.5	34.5	63.4	68.5		6.2	11.6	14.1	
Chemotherapy								< 0.001				< 0.001
Yes	1217	67.2	783	64.1	23.8	54.0	60.0		3.6	8.6	11.2	
No	594	32.8	438	35.9	38.7	61.8	64.6		10.4	16.3	18.6	
Radiotherapy								< 0.001				0.773
Yes	929	51.3	595	48.7	21.7	52.1	58.8		4.1	10.3	13.3	
No	882	48.7	626	51.3	36.0	61.2	64.4		7.6	12.0	13.8	

The median follow-up for these patients was 16 months (range: 7 to 33 months). During the follow-up period, 1221 patients died: 986 died of specific causes, and 235 died of other causes. The top three other causes of death were heart disease (27.2%), chronic obstructive pulmonary disease (COPD) and allied conditions (20.9%), and cerebrovascular diseases (4.7%).

### Probability of death

**Fig. 2:**
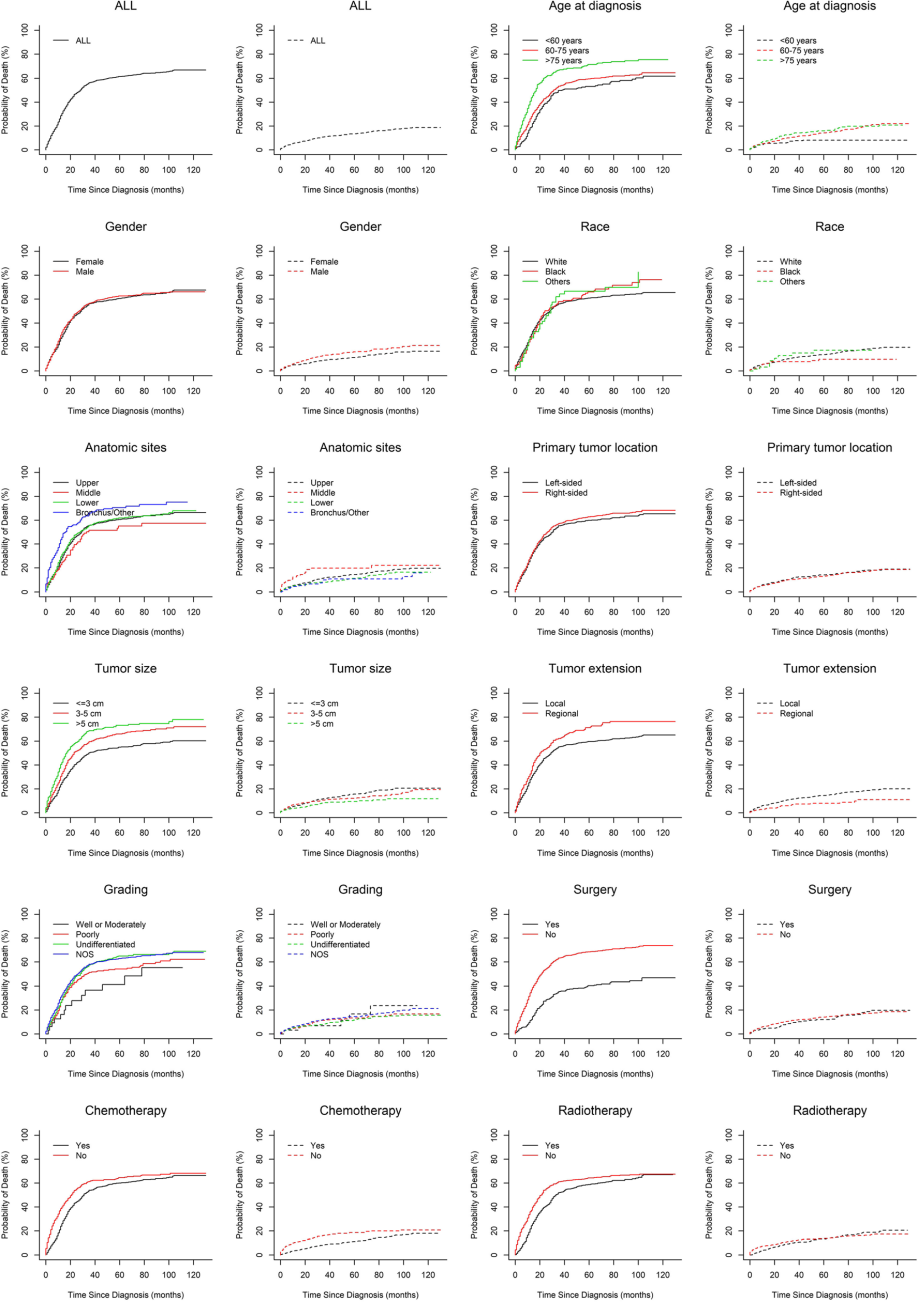
Cumulative incidence estimates of mortality of stage I SCLC patients by key characteristics (dotted line: death from other causes, solid line: cause-specific death)

The cumulative incidence function curves are plotted in Fig. 2. The one-, three-, and five-year estimates of the cumulative incidence of mortality according to the age at diagnosis, gender, race, anatomic sites, laterality, tumor size, tumor extension, grading, and treatment are summarized in Table 1. The five-year cumulative incidence of mortalities resulting from specific causes and other causes was 61.5 and 13.6%, respectively. Patients with the characteristics of big tumor size, regional tumor extension, older age, and no surgery, chemotherapy, and radiotherapy were associated with high cause-specific death probabilities. Patients aged more than 75 years had the highest probability of death resulting from specific causes (71.4%). The cumulative incidence of cause-specific death for patients who did not undergo surgery was as low as 40.4%. As for the patients who did not receive chemotherapy and radiotherapy, their cumulative incidence of cause-specific death was 64.6 and 64.4%, respectively.

Considering the non-linear effect of age and tumor size, we used restricted cubic splines to flexibly model continuous variables. We conducted the joint test to see whether the group of coefficients as a whole was statistically significant or not (*P* < 0.001). As the results of competing risk model displayed on Table 2, tumor size, extent of tumor, laterality of tumor, surgery, and radiotherapy could strongly predict cancer-specific death. Patients who underwent surgery or radiotherapy had a lower cause-specific mortality, with a subdistribution hazard ratios (sdHR) of 0.370 (95%CI 0.304–0.450) and 0.553 (95%CI 0.477–0.641), respectively. Patients with regional tumor extension were more likely to die of their disease, with an sdHR of 1.434 (95%CI 1.216–1.693), when compared with local extension. Additionally, right-sided and larger tumor size were also associated with worse cancer-specific outcomes. For those patients who died from other causes, age, male, local extension, and patients without chemotherapy had a more aggressive impact, with a higher sdHR.

**Table 2:** Proportional Subdistribution Hazard Models of Probabilities of Cancer-Specific Death and Death from Other Causes for Patients with Stage I SCLC

Characteristics	Cancer-Specific Death			Death from Other Causes		
	Coefficient	_**sd**_HR (95%CI)	P	Coefficient	_**sd**_HR (95%CI)	P
Age	0.011	1.010 (0.997–1.024)	0.130	0.056	1.057 (1.010–1.105)	0.015
Age’	0.015	1.015 (0.999–1.031)	0.065	−0.050	0.951 (0.911–0.993)	0.024
Tumor size	0.118	1.125 (1.013–1.249)	0.027	−0.106	0.899 (0.746–1.083)	0.260
Tumor size’	−0.071	0.931 (0.806–1.076)	0.340	0.044	1.045 (0.780–1.399)	0.770
Male	−0.024	0.976 (0.858–1.110)	0.720	0.332	1.393 (1.071–1.811)	0.013
Race						
Black	− 0.022	0.978 (0.805–1.187)	0.830	−0.454	0.634 (0.368–1.094)	0.100
Others	−0.159	0.853 (0.591–1.231)	0.400	−0.028	0.972 (0.490–1.929)	0.940
Anatomic sites						
Middle	−0.248	0.780 (0.580–1.050)	0.100	0.424	1.528 (0.937–2.492)	0.089
Lower	0.011	1.010 (0.874–1.168)	0.880	−0.256	0.774 (0.564–1.060)	0.110
Bronchus/Other	0.256	1.291 (1.018–1.636)	0.034	−0.192	0.825 (0.492–1.382)	0.470
Right-sided	0.149	1.160 (1.015–1.324)	0.028	−0.132	0.876 (0.669–1.147)	0.340
Regional	0.361	1.434 (1.216–1.693)	< 0.001	− 0.516	0.597 (0.381–0.934)	0.024
Grading						
Poorly	0.171	1.186 (0.701–2.006)	0.520	−0.213	0.807 (0.317–2.053)	0.650
Undifferentiated	0.229	1.257 (0.748–2.111)	0.390	−0.353	0.702 (0.277–1.779)	0.460
NOS	0.156	1.168 (0.698–1.955)	0.550	−0.108	0.897 (0.356–2.259)	0.820
Surgery	−0.992	0.370 (0.304–0.450)	< 0.001	−0.162	0.850 (0.590–1.223)	0.380
Chemotherapy	−0.064	0.937 (0.801–1.096)	0.420	−0.582	0.558 (0.411–0.758)	< 0.001
Radiotherapy	−0.592	0.553 (0.477–0.641)	< 0.001	0.182	1.199 (0.885–1.625)	0.240

Note: Age’ and Tumor size’ are constructed spline variables (when k = 3)

### Nomogram

**Fig. 3:**
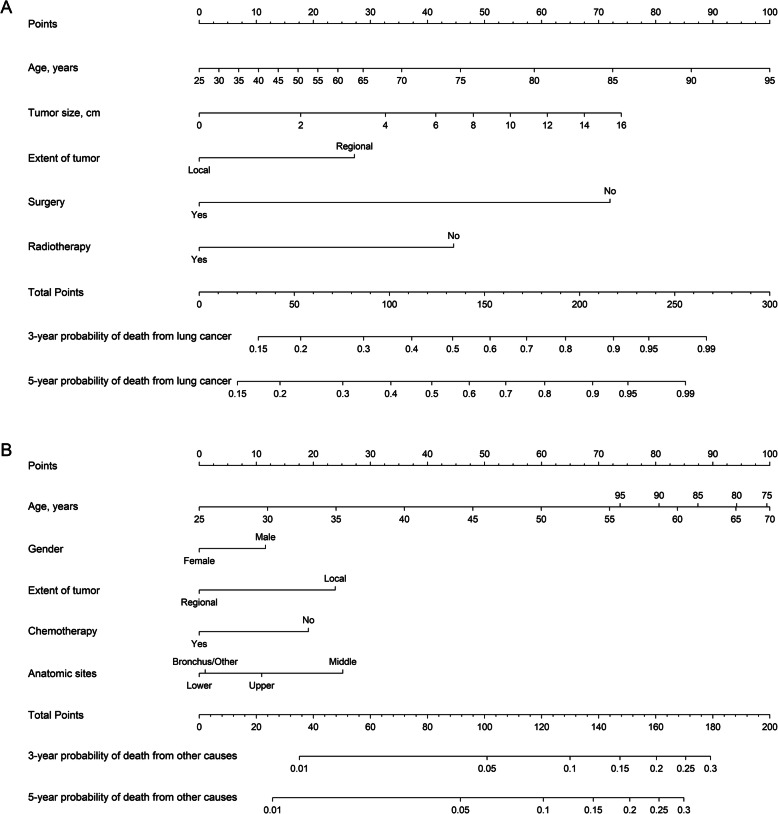
Nomogram to predict three- and five-year probabilities of mortality due to different causes for stage I SCLC patients: **a** cause-specific death and **b** death from other causes

The nomogram built on the basis of Fine and Gray’s model is shown in Fig. 3. The nomogram was used to find the corresponding score on the points row above the graph for each variable included in the model. All the assigned scores of the variables were added to obtain the total score, and then, a straight line was drawn to the bottom of the graph to estimate the probability of death.

### Model performance

**Fig. 4:**
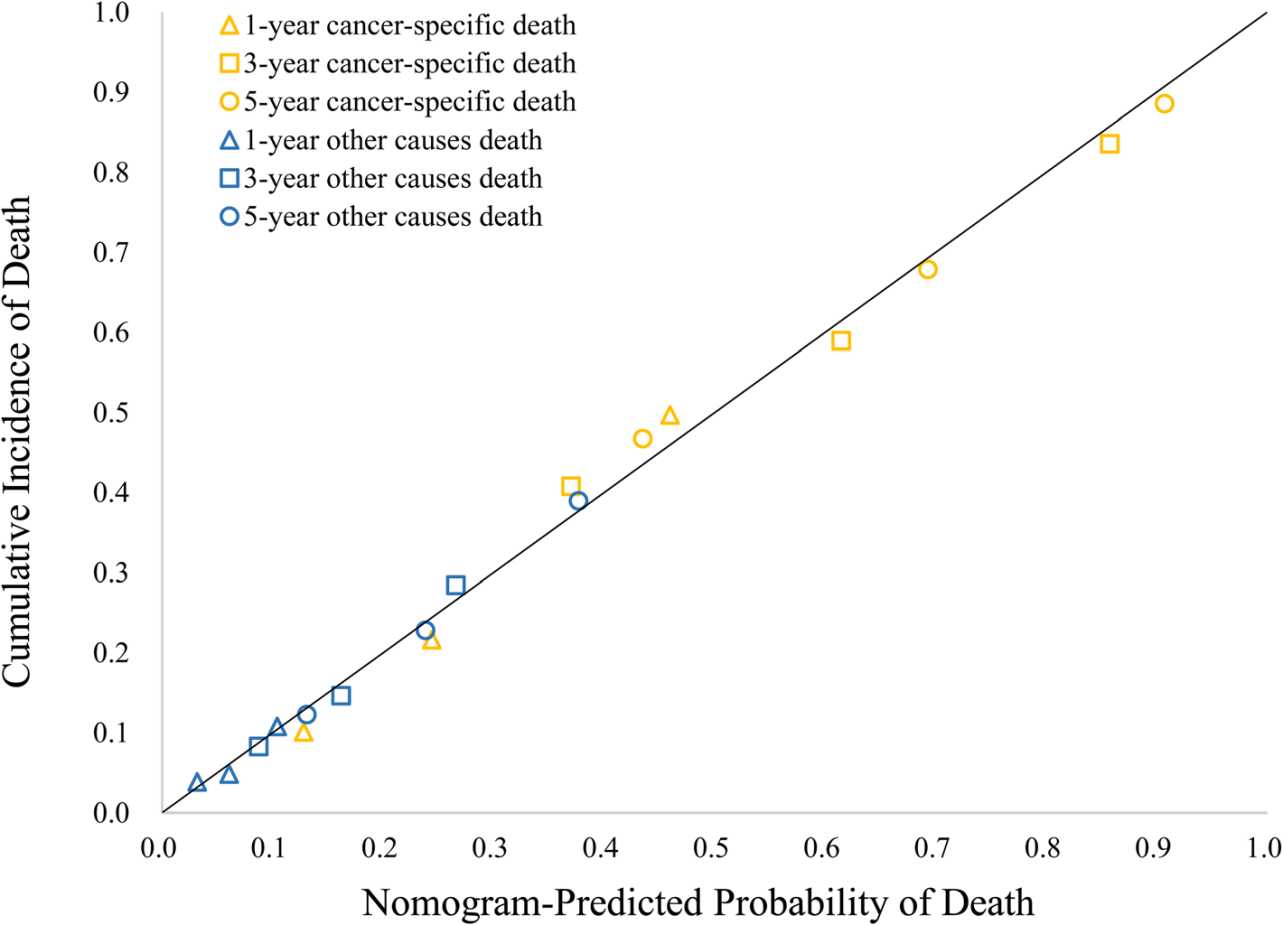
Calibration plot indicating the performance of the nomogram

The Harrell C index [5] was applied to indicate the discrimination, and a calibration plot obtained using the method provided by Gray [2], which was adopted to evaluate calibration. Discrimination, as measured by the 1000 resample bootstrap-corrected C index, was 0.696 (95% CI: 0.688–0.705) for the cancer-specific death and 0.672 (95% CI: 0.650–0.694) for other causes resulting in death. The calibration plot (Fig. 4) showed a high consistency between the predicted and the observed events.

## Discussion

In this study, we assessed the cumulative incidence of mortality resulting from different causes in stage I SCLC patients, who were a part of a large cohort considered in the SEER database. At the same time, we constructed a proportional sub-distribution model and a competing risk nomogram with variables to investigate the three- and five-year cause-specific mortality.

Previous study [11–16] showed that the five-year cumulative incidence rate in patients diagnosed with stage I SCLC who were instructed to undergo surgery was from 40 to 60%. A retrospective analysis from the SEER database showed that patients with stage I SCLC who underwent lobectomy had a higher 5-year survival of 50.3% [17]. In our study, the five-year cumulative incidence rates of cause-specific and other cause-related mortality were 61.5 and 13.6%, respectively, indicating that SCLC had a high mortality rate and poor prognosis. However, many patients died from other diseases despite the poor prognosis. With an increase in the age and the tumor size, the cumulative incidence of death resulting from all the causes gradually increased. The treatment of SCLC, including surgery, chemotherapy, and radiotherapy, diminished the cumulative incidence of mortality of all the causes. The regional extent of a tumor statistically increased the cumulative incidence, which indicated that the treatment of the limited early stage of cancer was beneficial to the patients’ prognosis. For example, a 70 years patient with tumor size of 4 cm and regional extent of tumor, receiving surgery and radiotherapy has an estimate of 3-year and 5-year probability of death due to lung cancer of 33.7 and 38.1%, respectively.

According to the present competing risk model, the predictors of cause-specific death for stage I SCLC included tumor size, extent of tumor, surgery, and radiotherapy. There was a high probability in patients with the characteristics of the regional extent of tumor, large tumor size, no surgery, or radiotherapy to die of SCLC. Gender did not affect the cause-specific mortality, but the male patients were more prone to dying from other causes. Age affected other cause-related SCLC mortality. Hence, it is important to take actions to prevent older patients from dying from other diseases irrespective of the SCLC treatment. We did not find any significant effects of race and laterality on cause-specific death and death from other causes. Anatomic sites and grading were only significant in the cases of cause-specific death. Wang et al. [18] developed a nomogram prognostic model for SCLC patients and validated the model using an independent patient cohort. Their nomogram performs better than earlier models, including those using AJCC staging. However, because of lacking the Stage I SCLC competing risk analyses in their model, we cannot compare the results between Wang’s model and our model in this study.

Patients diagnosed with SCLC without any lymph node metastasis at a very early stage may undergo surgical resection of the lesion as the initial treatment procedure. According to the National Comprehensive Cancer Network guidelines, postoperative chemotherapy is recommended for stage I SCLC rather than radiation. Our study showed that surgery could effectively reduce the number of cancer-specific deaths and that the one-year cumulative incidence dropped from 34.5 to 11.2%. Like surgery, chemotherapy and radiotherapy improved the one-year survival rate. It is necessary to consider radiation before or after surgery, and this needs more validation. As SCLC is characterized by rapid growth, high invasiveness, and early metastasis, the five-year cumulative incidence was relatively high irrespective of the form of treatment. Our results indicated that treatment did not benefit the five-year survival rate. Therefore, early diagnosis and treatment are very critical and can markedly improve the one-year survival rate.

It is undeniable that our prediction model has some limitations. First, approximately 27% of the patients in our study were diagnosed during 2012–2014, which resulted in relatively short follow-up time. We could expect that longer follow-up time may help to improve the accuracy of model prediction. Second, several treatment-related factors weren’t included in the model, such as the plans of chemotherapy, number of cycles, the doses and methods of radiotherapy and the follow up treatment after recurrence. These factors can also influence the prognosis. Third, our model only provides a reference to clinical doctors. More complicated clinical factors will also be taken into account in their treatment decisions. Fourth, the comorbidity was a significant factor when physicians deciding treatment strategies. It was indeed a limitation that we established a prognostic model without comorbidity information. But we considered other vital clinical characters which could be obtained in SEER database with large sample and we believed this model could also providing valuable implications in clinical practice for stage I SCLC patients.

## Conclusions

The cumulative incidence of mortality due to specific causes and other causes in stage I SCLC patients was calculated using a SEER database analysis. We also constructed the competing risk regression model for stage I SCLC and a competing risk nomogram to predict the three- and five-year cause-specific mortality individually. The nomogram could predict the prognosis conveniently and directly for stage I SCLC patients and help clinicians to make critical treatment decisions and choose appropriate strategies.

## Supplementary information

**Additional file 1.**

## Data Availability

Limited Use Agreement for Surveillance, Epidemiology, and End Results (SEER) Program (https://seer.cancer.gov) SEER*Stat Database: accession number (15586-Nov2016). The data can be used publicly.

## Abbreviations

SCLC: Small-cell lung cancer
CIFs: Cumulative incidence functions
NCCN: National Comprehensive Cancer Network
SEER: Surveillance, Epidemiology, and End Results
sdHR: Sub-distribution hazard ratio
COPD: Chronic obstructive pulmonary disease
NOS: Not otherwise specified
CI: Confidence interval
