# Supplementary material for: Nomogram model for predicting cause-specific mortality in patients with stage I small-cell lung cancer: a competing risk analysis
Source: BMC Cancer. 2020 Aug 24;20:793. doi: 10.1186/s12885-020-07271-9 (PMC7445928; doi:10.1186/s12885-020-07271-9)
Supplement: Supplementary file 1 — Additional file 1. [file 12885_2020_7271_MOESM1_ESM.docx]

Filename: Case Listing Session-1 Matrix-1
SEER*Stat Version: 8.3.6
Date: July 24, 2020

Session Type: Case Listing

SUGGESTED CITATION
Software: Surveillance Research Program, National Cancer Institute SEER*Stat software (www.seer.cancer.gov/seerstat) version 8.3.6.
Data: Surveillance, Epidemiology, and End Results (SEER) Program (www.seer.cancer.gov) SEER*Stat Database: Incidence - SEER 18 Regs Custom Data (with additional treatment fields), Nov 2018 Sub (1975-2016 varying).

- Linked To County Attributes - Total U.S., 1969-2017 Counties, National Cancer Institute, DCCPS, Surveillance Research Program, released April 2019, based on the November 2018 submission.

DATA
Database: Incidence - SEER 18 Regs Custom Data (with additional treatment fields), Nov 2018 Sub (1975-2016 varying). - Linked To County Attributes - Total U.S., 1969-2017 Counties


SELECTION
Select Only: Malignant Behavior, Known Age, Cases in Research Database

Case: {Site and Morphology.Primary Site - labeled} = 'C34.0-Main bronchus','C34.1-Upper lobe, lung','C34.2-Middle lobe, lung','C34.3-Lower lobe, lung','C34.8-Overlapping lesion of lung','C34.9-Lung, NOS'

AND {Site and Morphology.Laterality} = 'Right - origin of primary','Left - origin of primary'

AND {Site and Morphology.ICD-O-3 Hist/behav, malignant} = '8010/3: Carcinoma, NOS','8012/3: Large cell carcinoma, NOS','8013/3: Large cell neuroendocrine carcinoma','8014/3: Large cell carcinoma with rhabdoid phenotype','8015/3: Glassy cell carcinoma','8020/3: Carcinoma, undifferentiated, NOS','8021/3: Carcinoma, anaplastic, NOS','8022/3: Pleomorphic carcinoma','8030/3: Giant cell and spindle cell carcinoma','8031/3: Giant cell carcinoma','8032/3: Spindle cell carcinoma, NOS','8046/3: Non-small cell carcinoma','8050/3: Papillary carcinoma, NOS','8051/3: Verrucous carcinoma, NOS','8052/3: Papillary squamous cell carcinoma','8070/3: Squamous cell carcinoma, NOS','8071/3: Squamous cell carcinoma, keratinizing, NOS','8072/3: Squamous cell carcinoma, large cell, nonkeratinizing, NOS','8073/3: Squamous cell carcinoma, small cell, nonkeratinizing','8074/3: Squamous cell carcinoma, spindle cell','8075/3: Squamous cell carcinoma, adenoid','8076/3: Squamous cell carcinoma, micro-invasive','8077/3: Squamous cell carcinoma, grade III','8078/3: Squamous cell carcinoma with horn formation','8140/3: Adenocarcinoma, NOS','8141/3: Scirrhous adenocarcinoma','8142/3: Linitis plastica','8143/3: Superficial spreading adenocarcinoma','8144/3: Adenocarcinoma, intestinal type','8145/3: Carcinoma, diffuse type','8146/3: Monomorphic adenocarcinoma','8147/3: Basal cell adenocarcinoma','8250/3: Bronchiolo-alveolar adenocarcinoma, NOS','8251/3: Alveolar adenocarcinoma','8252/3: Bronchiolo-alveolar carcinoma, non-mucinous','8253/3: Bronchiolo-alveolar carcinoma, mucinous','8254/3: Bronchiolo-alveolar carcinoma, mixed mucinous and non-mucinous','8255/3: Adenocarcinoma with mixed subtypes','8260/3: Papillary adenocarcinoma, NOS','8310/3: Clear cell adenocarcinoma, NOS','8323/3: Mixed cell adenocarcinoma','8430/3: Mucoepidermoid carcinoma','8480/3: Mucinous adenocarcinoma','8481/3: Mucin-producing adenocarcinoma','8482/3: Mucinous adenocarcinoma, endocervical type','8490/3: Signet ring cell carcinoma','8550/3: Acinar cell carcinoma','8560/3: Adenosquamous carcinoma','8570/3: Adenocarcinoma with squamous metaplasia','8571/3: Adenocarcinoma with cartilaginous and osseous metaplasia','8572/3: Adenocarcinoma with spindle cell metaplasia','8573/3: Adenocarcinoma with apocrine metaplasia','8574/3: Adenocarcinoma with neuroendocrine differentiation','8575/3: Metaplastic carcinoma, NOS'

AND {Site and Morphology.Diagnostic Confirmation} = 'Microscopically confirmed'

AND {Stage - 6th edition.Derived AJCC Stage Group, 6th ed (2004-2015)} = 'I','INOS','IA','IA1','IA2','IB','IB1','IB2','IC','IS','IEA','IEB','IE','ISA','ISB','II','IINOS','IIA','IIB','IIC','IIEA','IIEB','IIE','IISA','IISB','IIS','IIESA','IIESB','IIES','III','IIINOS','IIIA','IIIB','IIIC','IIIEA','IIIEB','IIIE','IIISA','IIISB','IIIS','IIIESA','IIIESB','IIIES','IV','IVNOS','IVA','IVB','IVC'

AND {Multiple Primary Fields.Sequence number} = 'One primary only'

AND {Multiple Primary Fields.First malignant primary indicator} = 'Yes'

TABLE
Column: Sex; Primary Site-labeled; Laterality; Survival months; SEER cause-specific death classification; Age at diagnosis; Year of diagnosis; RX Summ-Surg Prim Site(1998+); Histologic Type ICD-0-3; Vital status recode(study cutoff used); Year of birth; First malignant primary indicator; Type of Reporting Source; Radiation recode; Reason no cancer-directed surgery; Radiation sequence with surgery; RX Summ-Scope Reg LN Sur(2003+); RX Summ-Surg Oth Reg/Dis(2003+); Chemotherapy recode(yes, no/unk); Race/ethnicity; Race recode(White, Black, Other); COD to site recode; Race recode(W,B,AI,API); Grade; Diagnostic Confirmation; Month of diagnosis; Regional nodes examined(1988+); Regional nodes positive(1988+); Derived AJCC Stage Group, 7th ed (2010-2015); Derived AJCC T, 7th ed (2010-2015); Derived AJCC N, 7th ed (2010-2015); Derived AJCC M, 7th ed (2010-2015); Derived AJCC Stage Group, 6th ed (2004-2015); Derived AJCC T, 6th ed (2004-2015); Derived AJCC N, 6th ed (2004-2015); Derived AJCC M, 6th ed (2004-2015); CS tumor size(2004-2015); CS extension(2004-2015); CS lymph nodes (2004-2015)
